# Nonemergency Medical Transportation Benefit in Traditional Medicare Advantage and Value-Based Plans

**DOI:** 10.1001/jamanetworkopen.2024.49038

**Published:** 2024-12-04

**Authors:** Yunrong Shen, Xin Hu, Ryan D. Nipp, K. Robin Yabroff, Arthur S. Hong, Joshua M. Liao, Changchuan Jiang

**Affiliations:** 1 R. Ken Coit College of Pharmacy, University of Arizona, Tucson; 2 Department of Radiation Oncology, Emory University School of Medicine, Atlanta, Georgia; 3 Department of Medicine, University of Oklahoma Health Sciences Center, Oklahoma City; 4 Surveillance and Health Equity Science Department, American Cancer Society, Atlanta, Georgia; 5 Division of Hematology and Oncology, Department of Internal Medicine, University of Texas Southwestern Medical Center, Dallas; 6 Peter O’Donnell Jr School of Public Health, University of Texas Southwestern Medical Center, Dallas

## Abstract

This cross-sectional study examines differences in the provision of nonemergency medical transportation benefits between traditional and value-based Medicare plans.

## Introduction

Lack of affordable and reliable transportation limits health care access for underserved populations with chronic illnesses.^1,2^ Nonemergency medical transportation (NEMT) health insurance benefits may directly address this barrier.^3^

Medicare Advantage (MA) plans, insuring half of all Medicare beneficiaries, increasingly offer NEMT benefits.^4^ While traditional MA plans can adopt NEMT benefits, the Centers for Medicare & Medicaid Services (CMS) implemented the value-based insurance design (VBID) model test among MA plans in 2017 to give plans more flexibility in providing supplemental benefits like NEMT, focusing on reducing access inequities.^5,6^ While previous studies examined the availability of NEMT benefits in MA plans, none have evaluated its generosity, and whether they differ by traditional vs VBID plan type.

## Methods

This cross-sectional study followed the Strengthening the Reporting of Observational Studies in Epidemiology (STROBE) reporting guideline. The UT Southwestern Human Research Protection Program determined this study nonhuman participants research and therefore exempt from informed consent and review. We used 2020 to 2024 CMS Plan Benefits Package and MA enrollment files data (years with VBID NEMT benefit for over 20 plans). To promote representativeness, we included only VBID and traditional MA plans with 50 or more enrollees. We compared the weighted percentage of plans offering any and unrestricted NEMT benefits (those with no cost-sharing, prior authorization, referrals, and trip limits to either plan-approved or any health care locations). Additionally, we analyzed the restrictions among plans with NEMT benefits by plan type. R version 4.3.2 (R Project for Statistical Computing) was used for analysis. Data were analyzed from March to June 2024.

## Results

We identified 594 unique VIBD MA plans and 7673 unique traditional MA plans from 2020 to 2024. The number of VBID plans increased rapidly over time (Table). During the study, NEMT benefits were more prevalent in VBID plans compared with traditional MA plans (1159 [100.0%] vs 10 669 [44.5%]), with unrestricted benefits more frequent in VBID plans (77 [6.6%] vs 335 [1.4%]).

**Table.** Medicare Advantage Plan Characteristics and Nonemergency Medical Transportation (NEMT) Benefits, 2020-2024

Characteristics	Plan type and year, No. (%)									
	2020^a^		2021		2022		2023		2024	
	VBID^b^	Traditional	VBID	Traditional	VBID	Traditional	VBID	Traditional	VBID	Traditional
No. of MA plans	51	4108	131	4534	212	4956	325	5160	440	5203
Types of MA plan^c^										
HMO	34 (66.7)	2569 (62.6)	98 (74.8)	2782 (59.4)	148 (69.8)	2782 (56.1)	194 (59.7)	2443 (47.3)	257 (58.4)	2253 (43.3)
HMO/POS	2 (3.9)	379 (9.2)	2 (1.5)	437 (9.6)	12 (5.7)	512 (10.3)	35 (10.8)	856 (16.6)	44 (10.0)	957 (18.4)
Local PPO	15 (29.4)	1160 (28.2)	31 (23.7)	1404 (31.0)	52 (24.5)	1662 (33.5)	96 (29.5)	1861 (36.1)	139 (31.6)	1993 (38.3)
Total No. of enrollees	297 040	27 162 603	895 909	29 964 794	2 471 026	32 164 918	3 712 826	34 201 024	4 060 039	36 898 244
Percentage of plans offering										
NEMT benefits (%)^d^	51 (100.0)	1653 (40.2)	131 (100.0)	1912 (42.2)	212 (100.0)	2281 (46.0)	325 (100.0)	2549 (49.4)	440 (100.0)	2274 (43.7)
Unrestricted NEMT benefits (%)^e^	3 (5.9)	22 (0.5)	1 (0.8)	32 (0.7)	23 (10.8)	48 (1.0)	29 (8.9)	114 (2.2)	21 (4.8)	119 (2.3)

Abbreviations: HMO, Health Maintenance Organization; HMO/POS, health maintenance organization with point of service option; MA, Medicare Advantage; PPO, preferred provider organization; VBID, Value-Based Insurance Design.

^a^ Second quarter data was used in 2020 due to the unavailability of 2020 third quarter data on the Center for Medicare & Medicaid Services website. Third quarter data was used for all other years.

^b^ Only the first benefit package was included in our analysis, if a single VBID plan included multiple benefit packages.

^c^ To promote representativeness, we included the 3 most common types of Medicare Advantage plans (HMO, HMO/POS, PPO) in the final analysis, while excluding other plans, such as employer-sponsored and dual-eligible plans, as they are not accessible to the general public.

^d^ NEMT with restriction: NEMT benefits requiring any copayments, coinsurance, authorization, referrals, deductibles, or providing limited number of trips to plan-approved or any health locations.

^e^ NEMT without restriction: NEMT benefits not requiring any copayments, coinsurance, authorization, referrals, or deductibles for its NEMT benefits, and providing unlimited trips to either plan-approved or any health locations.

Among plans offering NEMT benefits, VBID plans had fewer restrictions to NEMT benefits regarding copays (365 [31.5%] vs 6372 [59.7%]), providing unlimited trips to plan-approved health locations (586 [51.6%] vs 1899 [17.8%]), and any health locations (221 [19.1%] vs 145 [1.4%]) than traditional plans. While VBID plans required prior authorization less frequently than traditional MA plans (145 [33.9%] vs 982 [43.2%]) in 2024, this requirement fluctuated over the study period. Requirements for referrals, deductibles, and coinsurance were low in both plan types (in less than 15% in all years) (Figure).

**Figure.**
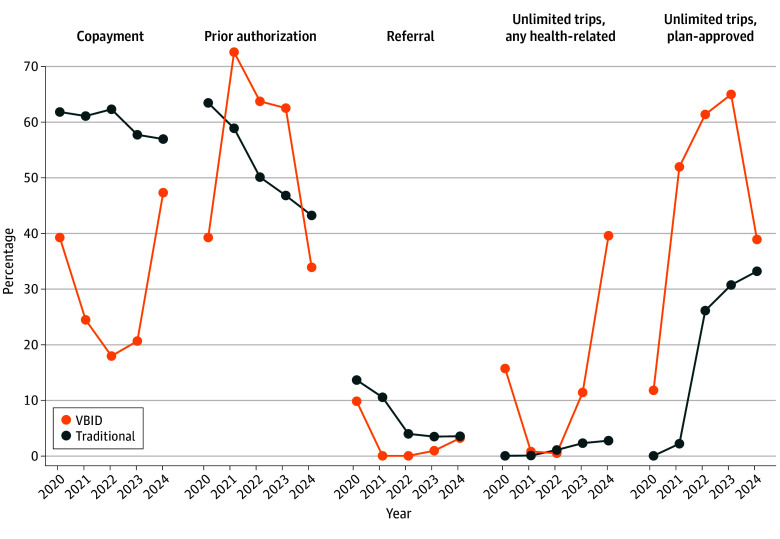
Cost-Sharing and Restrictions Among Value-Based Insurance Design and Traditional Medicare Advantage (MA) Plans With Nonemergency Medical Treatment (NEMT) Benefits, 2020-2024 Deductibles and coinsurance were required by neither value-based insurance design (VBID) nor traditional MA plans except for coinsurance required by only 1 of 4108 traditional MA plans in 2020 and 1 of 4534 in 2021. Due to the unavailability of 2020 third quarter data on the CMS website, we used second quarter data in 2020, while third quarter data was used for all other years. To promote representativeness, we included the 3 most common types of Medicare Advantage plans (Health Maintenance Organization [HMO], HMO with a point of service option, and preferred provider organization) in the final analysis, while excluding other plans, such as employer-sponsored and dual-eligible plans, as they are not accessible to the general public.

## Discussion

Our study found that VBID MA plans, compared with traditional MA plans, provided NEMT benefits and unrestricted benefits more frequently during 2020 to 2024. Among plans with such benefits, a smaller proportion of VBID plans had restrictions and cost-sharing in 2024. Recognizing the importance of addressing patient health-related social needs, including transportation barriers to care, better understanding of optimal benefit design is warranted to improve health equity.

Many patients, especially those with frequent health care needs, report that NEMT is crucial for their health.^1^ Lack of transportation is associated with increased emergency department use and mortality risk among patients with complex health conditions, such as cancer.^2^ Therefore, enhanced NEMT benefits under VBID and traditional MA models could bridge a key gap in health care delivery and outcomes, especially because over half of all Medicare beneficiaries were enrolled in MA plans. Starting from 2025, VBID plans will be required to offer supplemental benefits in at least 2 of 3 health-related social needs areas: food, transportation and housing insecurity, and/or living environment.^5^ Clinicians, facilities, and health care systems should work with patients to fully use these benefits.

Study limitations included a lack of details on cost-sharing and referral or prior authorization process by plan, patient-level data on benefit utilization, and association with health service use and outcomes. In conclusion, a higher proportion of VBID MA plans provided NEMT benefits with less restrictions compared with traditional MA plans, aligning with VBID’s goals of improving access to care and advancing health equity.

## Notes

Supplement.
Data Sharing Statement
