# Supplementary material for: Nonemergency Medical Transportation Benefit in Traditional Medicare Advantage and Value-Based Plans
Source: JAMA Netw Open. 2024 Dec 4;7(12):e2449038. doi: 10.1001/jamanetworkopen.2024.49038 (PMC11618458; doi:10.1001/jamanetworkopen.2024.49038)
Supplement: Supplement. — Data Sharing Statement [file jamanetwopen-e2449038-s001.pdf]

Data Sharing Statement

Shen. Nonemergency Medical Transportation Benefit in Traditional Medicare Advantage and Value-Based Plans. *JAMA Netw Open*. Published December 04, 2024.
doi:10.1001/jamanetworkopen.2024.49038

Data
Data available: Yes
Data types: Other (please specify)
Additional Information: Public available data has been used and available on CMS website
How to access data: Public available data has been used and available on CMS website
When available: With publication

Supporting Documents
Document types: None

Additional Information
Who can access the data: researchers whose proposed use of the data has been approved
Types of analyses: For any purpose
Mechanisms of data availability: With investigater support
